# Preperitoneal herniation after transabdominal preperitoneal patch plasty: a case report

**DOI:** 10.1093/jscr/rjad099

**Published:** 2023-03-09

**Authors:** Katsudai Shirakabe, Muramatsu Hirotada, Masaki Kanzaki

**Affiliations:** Department of General Surgery, Tokyo Bay Urayasu Ichikawa Medical Center, Urayasu, Japan; Department of General Surgery, Tokyo Bay Urayasu Ichikawa Medical Center, Urayasu, Japan; Department of General Surgery, Tokyo Bay Urayasu Ichikawa Medical Center, Urayasu, Japan

**Keywords:** hernia, laparoscopy, peritoneum, small bowel obstruction

## Abstract

Hernial repair is a common procedure performed by general surgeons. The use of laparoscopic hernial repair has recently increased; it was introduced in the 1980s, is minimally invasive and has few complications. However, because of this increase, rare complications that were previously unknown have been reported. A 63-year-old man underwent transabdominal preperitoneal patch plasty (TAPP) for a left inguinal hernia. Tension was felt in the peritoneum during peritoneal closure. On the fifth postoperative day, the patient was admitted for small bowel obstruction (SBO) and underwent reoperation; the closed peritoneum was lacerated, causing SBO because of herniation of the preperitoneal space. After the hernia was released, the peritoneum was closed again, and the surgery was completed. SBO after TAPP surgery is a rare complication. Several reports have shown staplers, barbed sutures and tacks causing SBO; this complication can be prevented with appropriate peritoneal closure techniques and treated with early laparoscopic surgery.

## INTRODUCTION

Hernia repair is currently the most frequently performed procedure by general surgeons. Ralph Ger [1] reported the first case of laparoscopic inguinal hernia repair. Several techniques have been proposed for treating inguinal hernias, including the Shouldice and Lichtenstein techniques, laparoscopic transabdominal preperitoneal patch plasty (TAPP) and total extraperitoneal patch plasty [2]. In recent years, the frequency of laparoscopic surgery for inguinal hernias has dramatically increased in many countries. It has been reported that laparoscopy enables faster recovery, a shorter time to return to work, reduced pain and a lower incidence of surgical site complications [2, 3].

Postoperative complications of laparoscopic surgery are rare and minor, including seroma formation, internal organ damage, chronic pain and testicular complications. As increasing numbers of TAPP procedures are being performed worldwide, uncommon complications have become more frequent and need to be considered in perioperative and postoperative management. Small bowel obstruction (SBO) is a rare complication after TAPP and has been reported to occur in ~0.1–0.23% of cases [4, 5]. This report presents a case of SBO caused by a laceration of the peritoneum after TAPP, resulting in a preperitoneal hernia.

## CASE REPORT

A 63-year-old male with a left inguinal hernia (Type L2) underwent TAPP. The mesh was fixed with a nonabsorbable Tucker. Peritoneal closure was achieved with continuous absorbable barbed sutures. During peritoneal closure, tension was present in the peritoneum; nevertheless, the procedure was completed. The operative time was one hour, and no intraoperative problems were observed. The patient had an uneventful postoperative course and was discharged on the second postoperative day; however, he was readmitted to the hospital on the fifth postoperative day because of SBO.

**Figure 1:**
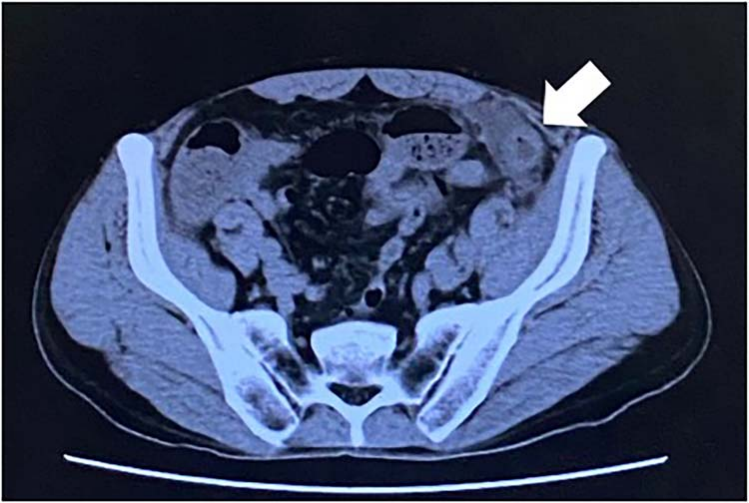
The white arrow shows the site of caliber change in the small bowel (axial section image).

**Figure 2:**
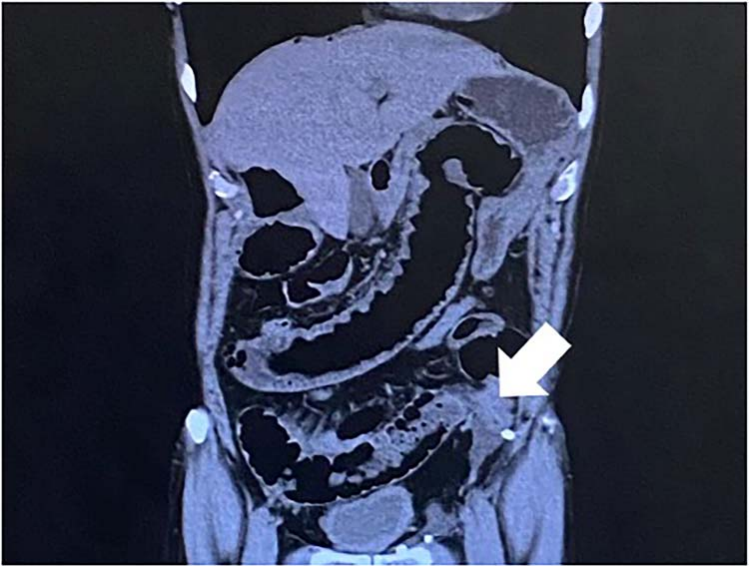
The white arrow shows the small bowel incarcerating into the preperitoneal space (coronal section image).

**Figure 3:**
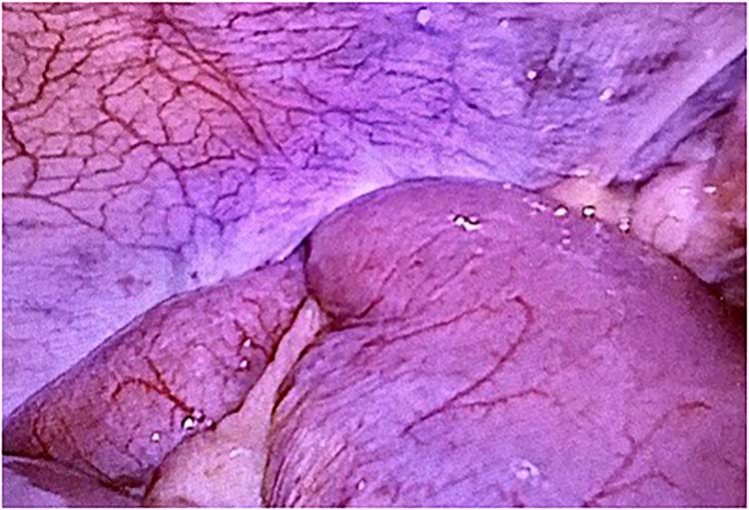
The closed peritoneum was lacerated, and the small bowel was incarcerated in the preperitoneal space.

**Figure 4:**
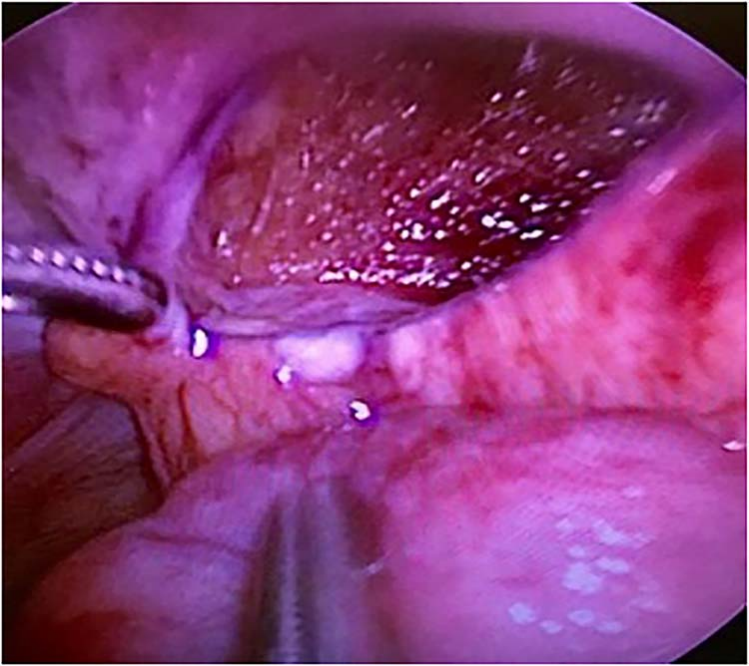
The lacerated peritoneal hole formed a hernia orifice.

Computed tomography revealed caliber changes in the small bowel at the site of post-hernia repair in the left inguinal region (Figs 1 and 2), and the patient underwent laparoscopic surgery under general anesthesia. Intraperitoneal observation revealed that the peritoneal suture in the left inguinal region was detached, and a hole in the peritoneum had formed a hernial orifice (Fig. 4), causing SBO because of preperitoneal herniation (Fig. 3). The hernia was released, the peritoneal hole was sutured again and the surgery was completed. The postoperative course was good, and the patient was discharged from the hospital on the third postoperative day after reoperation.

## DISCUSSION

A hernia is a protrusion of a viscus or part of the viscus through the cavity wall in which it normally resides. Conditions such as coughing, straining, obesity and intra-abdominal malignancy can precipitate hernias [6]. More than 20 million patients worldwide are treated for inguinal hernias annually [7]. Ger [1] reported the first case of laparoscopic inguinal hernia repair. Although laparoscopic hernia repair requires a longer operative time and depends on the surgeon’s expertise, it offers advantages such as less postoperative pain, fewer postoperative complications, shorter hospital stay and shorter disability period [8]. Recently, the types of laparoscopic surgeries for treating inguinal hernias have increased and are expected to grow further. Furthermore, the overall complication rate of TAPP repair is decreasing owing to technological advances. Statistics show that the incidence of SBO after TAPP repair is ~0.1–0.23% [4, 5].

There are various methods of perineal closure, including the use of staplers, tacks and barbed sutures, which largely depend on surgeon preference. SBO after TAPP repair is usually caused by peritoneal closure, herniation at the trocar site or barbed sutures [5, 9, 10].

Kapiris *et al*. [5] observed seven cases (0.23%) of SBO because of peritoneal herniation in a 7-year study of 3017 patients undergoing TAPP hernia repair. They found that peritoneal closure with sutures, compared with closure with staplers, decreased the incidence of this complication; this is probably the result of less tension on the peritoneal flap and a reduced risk of laceration. However, suture closure cannot completely eliminate this complication.

A preperitoneal herniation is an extremely rare condition that could be avoided. Adequate and skillful peritoneal closure, performed using a strictly standardized procedure, can achieve good results. Complete closure of the peritoneum during TAPP repair is essential for preventing adhesions and SBO because of mesh exposure.

Surgical techniques that reduce peritoneal tension by lowering the pneumoperitoneal pressure to 8–10 mmHg when closing the peritoneal flap and dissecting more peritoneum from the cord structures to allow closure are also important. If the defect is significant and suture closure is difficult, there have been reports of filling the defect with Surgicel Nu-Knit to eliminate dead space and prevent reincarceration [11].

Laparoscopic surgery is recommended for treating intestinal obstruction after TAPP surgery and in cases where this condition is anticipated. Although great care must be taken during port insertion because of dilatation of the bowel, we believe that laparoscopy is feasible.

Therefore, it is imperative to examine the activity of the entrapped bowel, and it should be performed with caution. If intestinal necrosis or perforation develops, bowel resection and mesh removal should be considered. Early diagnosis and appropriate treatment are crucial for successful outcomes in patients with SBO after TAPP.

In conclusion, although laparoscopic TAPP is widely used and accepted as a treatment for inguinal hernias, it is important to remember that rare complications can occur. Appropriate peritoneal closure is recommended to reduce the risk of intestinal hernias and bowel obstruction. We consider laparoscopy the procedure of choice to diagnose and treat this complication.
